# TRAPPC9 deficiency’s implication in “secondary” autism spectrum disorders

**DOI:** 10.1192/j.eurpsy.2023.1089

**Published:** 2023-07-19

**Authors:** F. Majdoub, A. Bouzid, A. Souissi, I. Boujelbene, W. Bouchaala, F. Kamoun, M. Ben said, C. Triki, S. Masmoudi, I. Ben Ayed

**Affiliations:** 1 Medical Genetics Department, University Hedi Chaker Hospital of Sfax, Tunisia., Medical school of Sfax, Tunisia; 2 Laboratory of Molecular and Cellular Screening Processes (LPCMC), Center of Biotechnology of Sfax, University of Sfax; 3 Child Neurology Department, University Hedi Chaker Hospital of Sfax, Medical school of Sfax, Tunisia, Sfax, Tunisia

## Abstract

**Introduction:**

Autism spectrum disorder (ASD) is a highly heterogeneous neurodevelopmental disorder with many contributing risk genes. Multiple intellectual disability (ID) susceptibility genes have been identified in ASDs to date. The trafficking protein particle complex subunit 9 *TRAPPC9* (OMIM#611966) in an autosomal recessive intellectual disability (ID) gene associated with not fully penetrant phenotype combining secondary microcephaly, dysmorphic facial features, obesity, autism spectrum disorder (ASD) and attention-deficit hyperactivity disorder (ADHD).

**Objectives:**

The aim of this study is to consider *TRAPPC9* deficiency in autosomal recessive ID with ASD.

**Methods:**

We present the observation of two siblings, born to Tunisian consanguineous and healthy parents, followed up for syndromic intellectual disability (ID) associated ASD and microcephaly. A clinical exome sequencing was performed to one child using a Trusight One kit of Illumina. We used sanger sequencing to validate the suspected variant for the other child and to specify the parental segregation.

**Results:**

A homozygous pathogenic variant in the *TRAPPC9* (NM_001160372.4) gene, c.1414C > T (p. Arg472Ter) were identified in one child. Sanger sequencing confirmed the homozygosity profile of this variant for the other child while the parents were both heterozygous carriers.

**Conclusions:**

Repetitive behaviours, especially hand-flapping, were the mean ASD feature in our patients. The current variant is known in the Tunisian population. It is described to lead to the creation of a premature stop codon and a truncating protein causing a *TRAPPC9* deficiency. The impairing neuronal NFkB signalling due to *TRAPPC9* deficiency has been suggested to be implicated in ASD. Due to the profound ID seen in both patients, we suggest the classification of ASD related to *TRAPPC9* deficiency as “secondary” rather than “primary”.

Our results support the implication of *TRAPPC9* in secondary ASD and shed the light on the possibility of screening p. Arg472Ter in Tunisian patients with this form of ASD as it is a recurrent mutation in the Tunisian population.

**Disclosure of Interest:**

None Declared

